# Facile biogenic fabrication of hydroxyapatite nanorods using cuttlefish bone and their bactericidal and biocompatibility study

**DOI:** 10.3762/bjnano.11.21

**Published:** 2020-02-04

**Authors:** Satheeshkumar Balu, Manisha Vidyavathy Sundaradoss, Swetha Andra, Jaison Jeevanandam

**Affiliations:** 1 Department of Ceramic Technology, Alagappa College of Technology, Anna University, Chennai 600025, India; 2 Department of Textile Technology, Alagappa College of Technology, Anna University, Chennai 600025, India; 3 Department of Chemical Engineering, Curtin University, Miri, Sarawak 98009, Malaysia

**Keywords:** antibacterial activity, biocompatibility, bone implant, cuttlefish bone, hard tissue treatment, hydroxyapatite, nanorods

## Abstract

Cuttlefish bones are an inexpensive source of calcium carbonate, which are produced in large amounts by the marine food industry, leading to environmental contamination and waste. The nontoxicity, worldwide availability and low production cost of cuttlefish bone products makes them an excellent calcium carbonate precursor for the fabrication of hydroxyapatite. In the present study, a novel oil-bath-mediated precipitation method was introduced for the synthesis of hydroxyapatite (Hap) nanorods using cuttlefish bone powder as a precursor (CB-Hap NRs). The obtained CB-Hap NRs were investigated using transmission electron microscopy (TEM), Fourier transform infrared spectroscopy (FTIR), X-ray diffraction (XRD) and thermogravimetric analysis (TGA) techniques to evaluate their physicochemical properties. The crystallite size (20.86 nm) obtained from XRD data and the elemental analysis (Ca/P molar ratio was estimated to be 1.6) showed that the Hap NRs are similar to that of natural human bone (≈1.67). Moreover, the FTIR data confirmed the presence of phosphate as a functional group and the TGA data revealed the thermal stability of Hap NRs. In addition, the antibacterial study showed a significant inhibitory effect of CB-Hap NRs against *S. aureus* (zone of inhibition – 14.5 ± 0.5 mm) and *E. coli* (13 ± 0.5 mm), whereas the blood compatibility test showed that the CB-Hap NRs exhibited a concentration-mediated hemolytic effect. These biogenic CB-Hap NRs with improved physicochemical properties, blood compatibility and antibacterial efficacy could be highly beneficial for orthopedic applications in the future.

## Introduction

Generally, the hard tissue of humans and animals, such as bone and teeth, are composed of natural hydroxyapatite (Hap), which is a bioactive ceramic material with high calcium phosphate concentration whereby the material can encounter damage or loss during an accident, infection, or trauma [1]. Because synthetic Hap exhibits similar chemical and biological properties to that of natural hard tissue, it is widely investigated for various orthopedic and dental treatments to restore damaged hard tissue [2]. It was reported in the literature that synthetic and conventional Hap has been prepared by various chemical procedures such as hydrothermal, sol–gel, mechanochemical, reverse microemulsion and precipitation methods, and the resulting material has been proposed to be highly beneficial in hard tissue treatments [3–5]. However, the hazardous chemicals involved in the synthesis process, which are toxic to healthy cells, is the primary limitation to employ these materials in widespread biomedical and environmental applications. Thus, the nontoxic, ecologically friendly preparation of Hap has received considerable attention in the bone and dental implant field. However, the development of a novel biomaterial for hard tissue treatments is still a major challenge due to the high material cost and lack of biocompatibility. Moreover, a highly biocompatible material, such as calcium phosphate, is required to overcome the increasing demand of biomaterials for hard tissue repair [6–7]. It is noteworthy that marine species, including corals, crabs, and fish bones, possess natural calcium phosphate and are currently being extracted and utilized as drug delivery carriers, tissue engineering scaffolds and dental cements in the biomedical field [8–9]. Hence, natural calcium phosphate from marine organisms plays a major role in recent developments of Hap for use in biomedical applications.

Cuttlefish (*Sepia officinalis*) is an important marine food that is available for human consumption, and tons of cuttlefish bones are produced as waste material every day by the marine food industry across the world, resulting in environmental contamination [10]. In ancient times, cuttlefish bone powder was used as biomedicine in China and India for oral health and is proven to be completely suitable for biomedical applications [11]. Further, the inorganic part of cuttlefish bone contains calcium carbonate (CaCO_3_) in the form of aragonite, along with other minerals such as sodium, magnesium, and strontium as trace elements, and these minerals play a substantial role in the bone healing process [12–13]. The main advantage of using cuttlefish bone powder to prepare Hap is their cost effectiveness and ecologically friendly nature, high availability, enhanced interconnectivity, and biocompatibility [14].

Recently, it was reported that the bonding ability of the scaffold with surrounding tissues is determined by the porosity of the material and that cuttlefish-bone-derived Hap has a superior porous structure [15]. This porous structure allows the blood vessels, which grow inside the biomaterial, to receive the required minerals and oxygen [16]. Additionally, the porous morphology in nanometer-sized Hap provides unique properties, such as high drug loading capacity and slow drug release in drug delivery systems for progressive advancement in osteoporosis and bone tumor treatments [17]. Various studies have reported the synthesis procedure of Hap nanoparticles from annealed cuttlefish bone using a hydrothermal method, which yields calcium oxide (CaO), and analyzed their physical and biological properties [18–20]. However, the hydrothermal method requires expensive autoclaves to maintain high temperature and pressure and uses toxic chemicals to yield different morphologies of Hap, especially nanorods [21]. These Hap nanorods possess enhanced porosity and high surface-to-volume ratio when compared to spherical nanometer-sized Hap particles, which facilitates interconnectivity and improves targeted cell internalization efficiency [22]. Thus, the aim of the present work is to use a simple, facile, unique, safe and cost-effective precipitation setup via an oil bath approach to synthesize Hap nanorods from cuttlefish bone powders and optimize their synthesis parameters. The systematic characterization using X-ray diffraction (XRD), Fourier transform infrared spectroscopy (FTIR) and transmission electron microscopy (TEM) was performed to confirm the presence of nanorods and to evaluate their physicochemical properties. Furthermore, in vitro antimicrobial studies with gram-positive (*Staphylococcus aureus*) and gram-negative (*Escherichia coli*) bacteria, in addition to hemolysis studies with healthy human blood were performed to evaluate the antibacterial and biocompatible efficacy of the cuttlefish bone powder derived Hap nanorods.

## Results and Discussion

### Crystallinity and phase formation analysis

**Figure 1:**
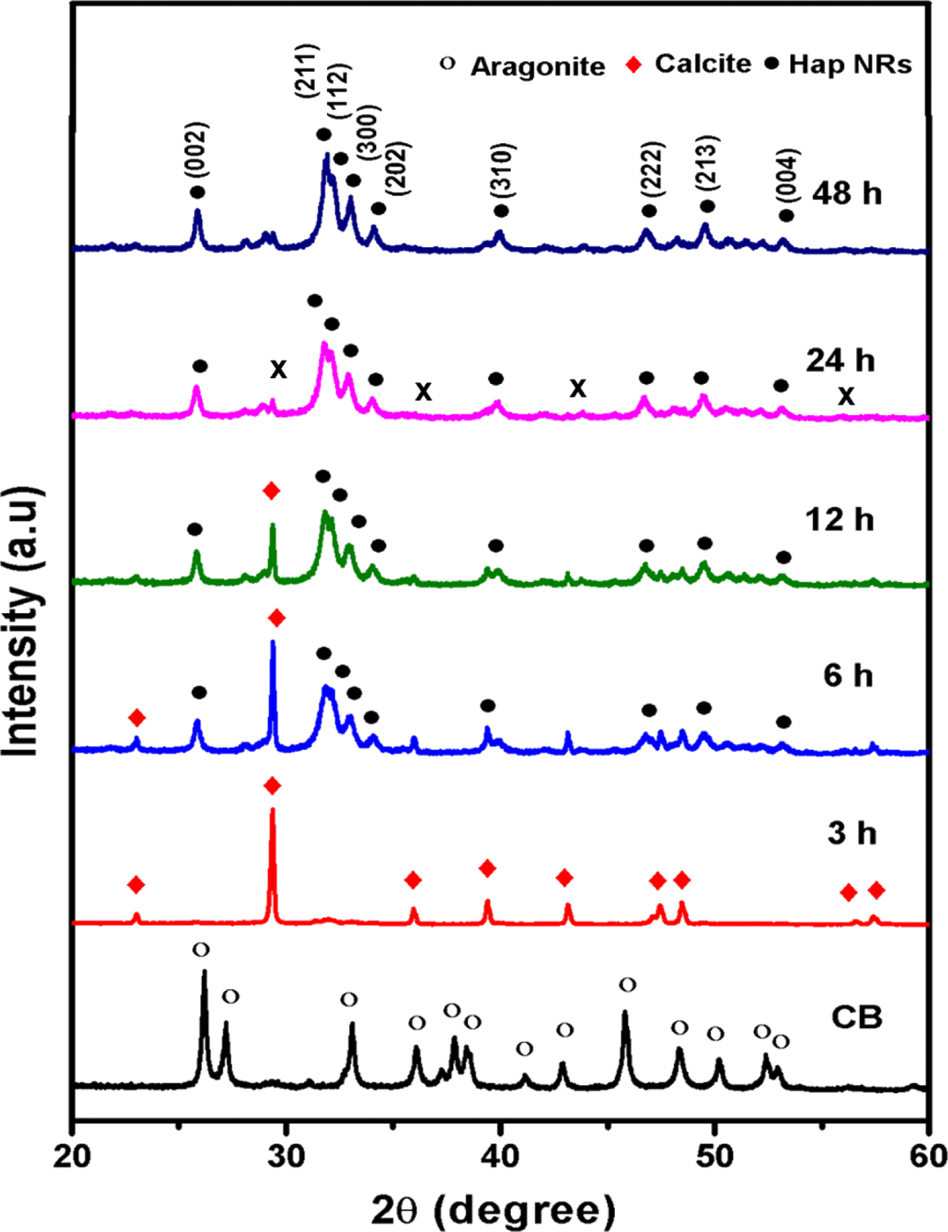
XRD spectrum of aragonite cuttlefish bone (CB), calcite, and hydroxyapatite (Hap) nanorods using cuttlefish bone powder as a precursor (CB-Hap NRs). The impurity peaks are indicated by an “x”.

The products obtained at various reaction times and the raw cuttlefish bone (CB) powders were characterized using powder XRD, revealing that a prolonged reaction time leads to the formation of highly crystalline Hap nanorods as shown in Figure 1. It is noteworthy that the XRD pattern of CB powder exactly matches with the aragonite crystal structure of calcium carbonate (CaCO_3_, JCPDS file 75-2230). Interestingly, aragonite CaCO_3_ was completely transformed into calcite CaCO_3_ (JCPDS file 71-2396) after 3 h of reaction time. The reflection peaks observed at 23°, 29°, 35°, 39°, 43°, 47°, 48°, 56° and 57° correspond to the (012), (104), (110), (113), (202), (018), (116), (211), and (122) planes, which indicates the rhombohedral structure of calcite [23]. Thus, it is evident that the reaction at basic pH conditions and a temperature of 80 °C supports the relaxation of aragonite calcium carbonate bonds to form calcite structure in 3 h [24]. It can be noted from the diffraction peaks that the formation of Hap crystal growth was initiated at 6 h of reaction time along with certain traces of calcite. Moreover, the intensity of the calcite at peak positions of 23° and 29° significantly decreased with increasing reaction time. This reveals that prolonged heating of calcite crystal relaxes the lattice of calcite to facilitate the formation of Hap [25]. Then, the calcite peak disappeared due to the complete formation of Hap at 24 h and 48 h of reaction time. The diffraction peaks were recorded at 25°, 31°, 32°, 33°, 34°, 39°, 46°, 49° and 53°, corresponding to the (002), (211), (112), (300), (202), (310), (222), (213), and (004) planes, which suggest that a hexagonal structure of Hap was formed. Even though the XRD peaks of Hap were formed at 24 h of reaction time stable crystallinity was observed. There also exists a certain minor impurity peak at 30°, 35°, 43° and 57°, which affects the purity. Thus, the reaction time was increased further to 48 h, which yields a complete stable crystal of Hap and is confirmed by the XRD peaks in Figure 1. The average crystallite size of the CB Hap is 20.86 nm (measured using full width half maximum of XRD data and the Scherrer equation), which is similar to the crystallite size of natural bone, and the lattice strain was 0.0055 nm.

### Evaluation of functional groups

**Figure 2:**
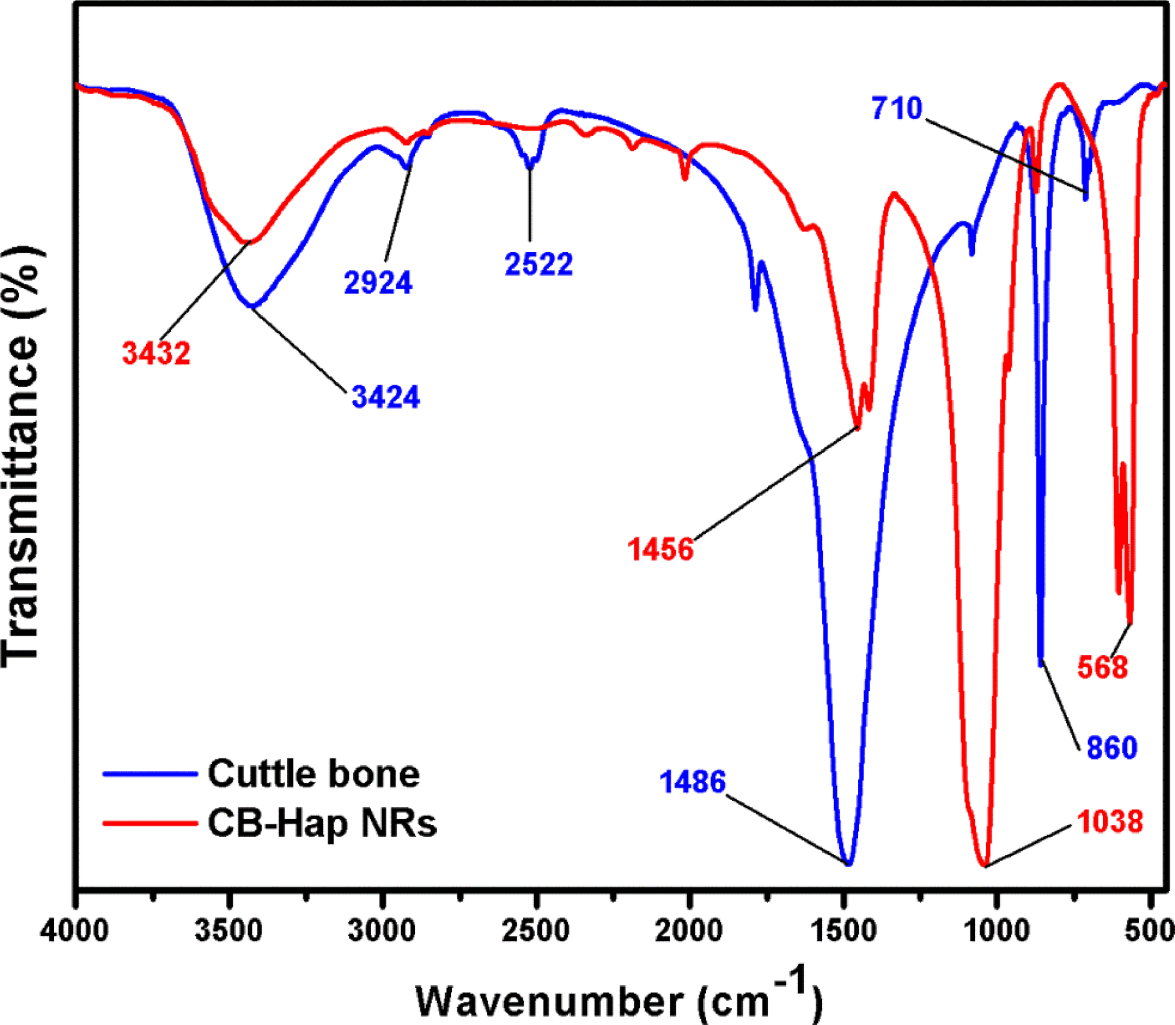
FTIR spectra of cuttlefish bone and hydroxyapatite (Hap) nanorods using cuttlefish bone powder as a precursor (CB-Hap NRs).

The FTIR spectra of CB and CB-Hap (48 h) with high crystallinity are shown in Figure 2, which is essential to analyze their functional groups. The FTIR spectral bands were recorded at 710, 860, 1486, 2522, and 2924 cm^−1^ for CB which indicates the existence of aragonite carbonates [26]. The presence of a characteristic peak at 3424 cm^−1^ is due to the vibration of O–H stretching, which indicates the presence of alcoholic functional groups [27]. Likewise, the IR bands recorded for the CB-Hap sample at 1038 and 568 cm^−1^ reveals the presence of functional asymmetric and symmetric PO_4_^3−^ stretching groups, respectively, which helps in the confirmation of the CB-Hap nanorod formation. Earlier reports suggest that there are two classes of carbonate substitutions on Hap, such as A-type, where the carbonates are substituted at the site of O–H vibrations [28], and B-type, where the substitution takes place at phosphate vibrational sites [29]. Hence, the band at 1456 cm^−1^ indicates the occupancy of B-type carbonates and the carbonated n-Hap are highly beneficial in biomedical applications, especially in hard tissue repair [30–31].

### Thermogravimetric analysis

**Figure 3:**
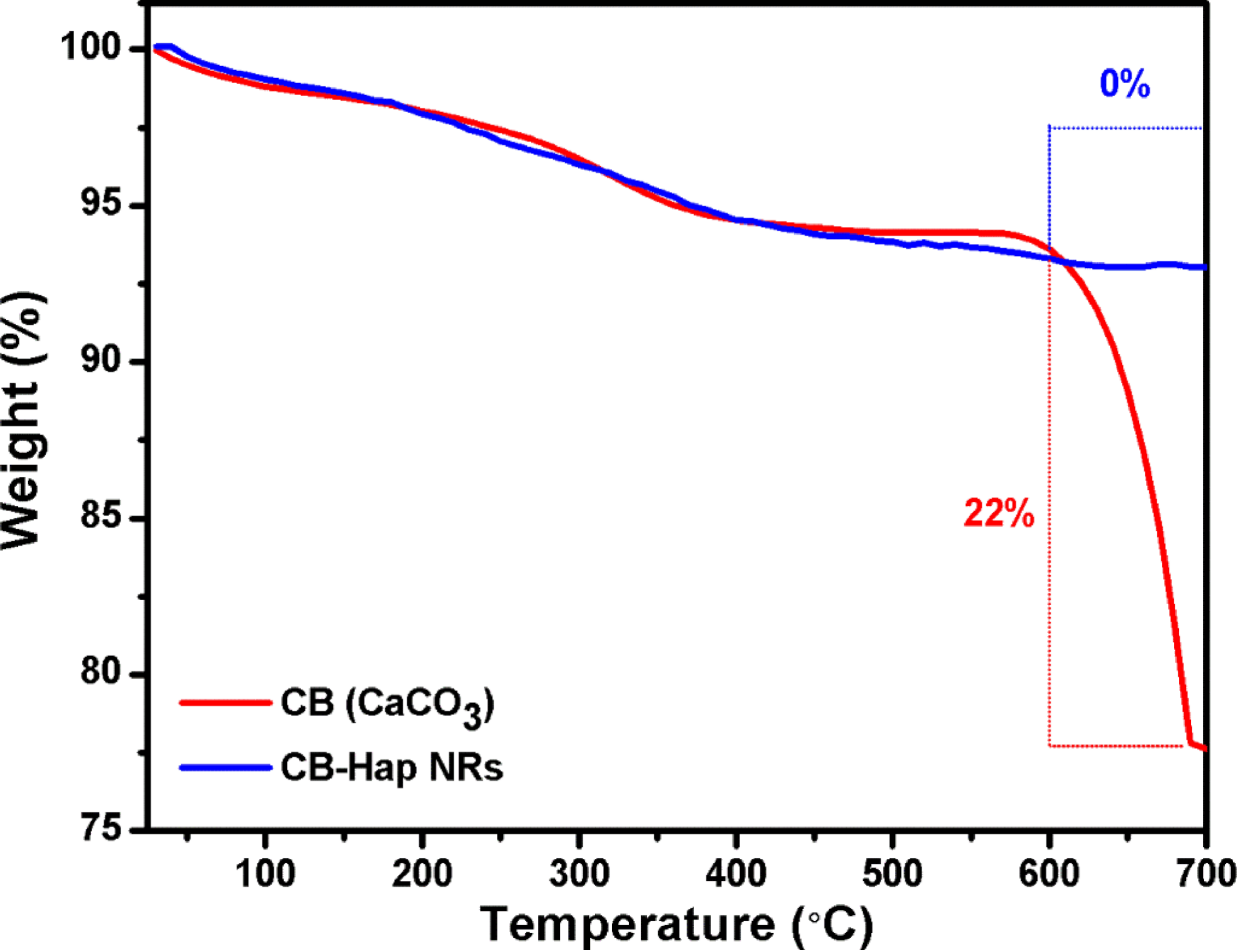
Thermogravimetric analysis (TGA) of cuttlefish bone (CB) powder and hydroxyapatite (Hap) nanorods using cuttlefish bone powder as a precursor (CB-Hap NRs).

Figure 3 shows the thermogravimetric analysis (TGA) of cuttlefish bone and CB-Hap nanorod powder. Both CB and CB-Hap NRs show a similar gradual weight loss of 6% from 38 to 583 °C due to the evaporation of water and organic substances of cuttlefish [32]. A drastic weight loss of 22% was observed from 583 to 689 °C for CB due to the decomposition of CaCO_3_, however no weight loss was observed for CB-Hap nanorods due to their thermal stability [33]. Herradi et al. (2017) suggested that a temperature above 750 °C is not suitable for Hap powder fabrication as a phase transformation is observed from 750 °C to 1000 °C [34]. Likewise, Venkatesan and Kim (2010) isolated Hap from *Thunnus obesus* fish and their study revealed that a high temperature facilitates aggregation and increases the crystal size of the nanoparticles [35]. On the other hand, it was proven by Hung et al. (2012) that an increase in the crystallite size will lead to reduction in the mechanical properties of the Hap nanoparticles [36]. Moreover, Ooi et al. (2018) recently reported that a high annealing temperature will affect the porous structure of Hap nanoparticles [37]. In the present study, the TGA (Figure 3) shows 0% weight loss at 600 °C to form CB-Hap NRs due to the complete removal of organic substances and water. Thus, 700 °C is selected as the optimum annealing temperature for the formation of Hap NRs as suggested from previous studies.

### Morphology and elemental analysis

**Figure 4:**
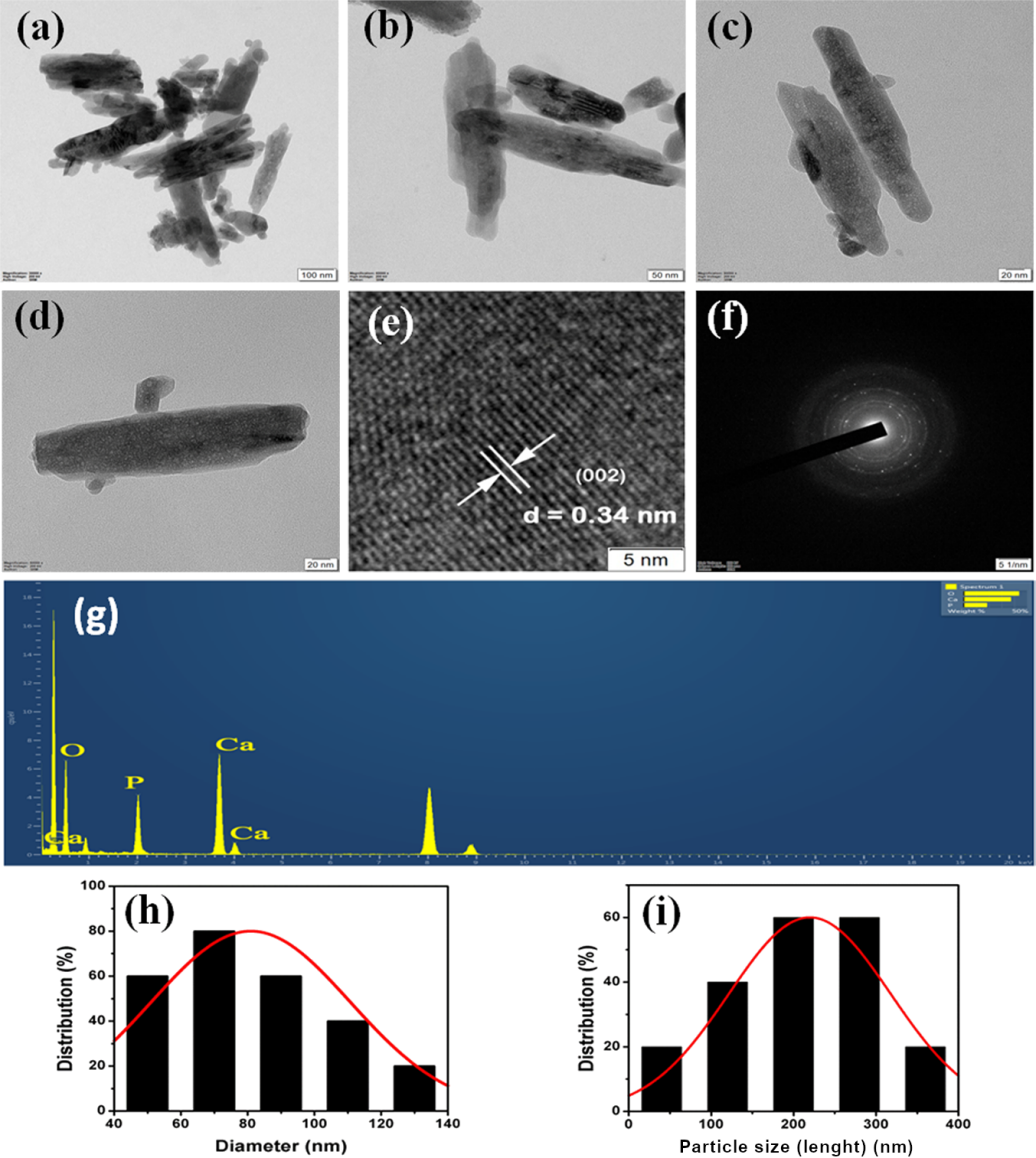
TEM images of hydroxyapatite (Hap) nanorods using cuttlefish bone powder as a precursor (CB-Hap NRs) at (a–d) different magnifications. (e) Calculation of d-spacing of CB-Hap NRs. (f) SAED pattern of CB-Hap NRs. (g) Elemental analysis of CB-Hap NRs and distribution of the (h) width and (i) length of CB-Hap NRs.

TEM micrographs of CB-derived Hap nanorods that are synthesized at a temperature of 80 °C and a reaction time of 48 h are shown in Figure 4a–d, which reveals their morphology and size. It is evident from the TEM micrographs at different magnifications that the morphology of n-Hap is rod-shaped. The average width and length of the nanorods was calculated as 79.05 ± 0.453 nm and 219.66 ± 0.38 nm, respectively, derived from the TEM micrographs using ImageJ software, as shown in Figure 4h,i. Kumar et al. (2015) synthesized Hap nanorods with 40–60 nm width and 500 to 700 nm length using waste shells of snail via a microwave irradiation method [38]. Similarly, Padmanabhan et al. (2009) prepared the Hap NRs using the sol–gel method that had 70–90 nm width and 400–500 nm length [39]. Likewise, Papageorgiou et al. (2014) fabricated Hap NRs with 20 nm width and 400 nm length by the hydrothermal method [40]. Thus, it is evident from the previous studies that the synthesis approach plays a crucial role in altering the size of the Hap NRs, which eventually determines their biological properties [41–42]. In addition, Ivankovic et al. (2009) prepared porous plate and needle-like Hap nanoparticles using cuttlefish bone with a hydrothermal approach [32]. However, the Hap NRs in the present work are nonporous as observed in the TEM images, which may affect the biocompatibility, while utilizing them as scaffolds. Hence, the calcination temperature must be optimized in the future studies to provide porosity in the Hap NRs to improving their biocompatibility with tissue. Further, Jadalannagari et al. (2011) and Zanotto et al. (2012) synthesized Hap NRs with smaller width and length via sol–gel and precipitation approaches, respectively [43–44], as compared to the present study. However, it is noteworthy that the biogenic fabrication of nanometer-sized Hap rods (even though larger in size) as mentioned in this study can help to yield less toxic Hap NRs (refer section “Blood compatibility analysis”), which will be beneficial for biomedical applications, when compared to the non-biogenic synthesized Hap NRs that uses toxic chemicals and lacks ability to control morphology [45–47]. Furthermore, the d-spacing was measured using a Gatan digital micrograph and was found to be around 0.34 nm, which corresponds to the (002) plane as shown in Figure 4e. Additionally, the interplanar distance was determined by the diffraction ring pattern as shown in Figure 4f and was estimated as 0.191, 0.722, and 0.122 nm, which is consistent with the hexagonal structure of Hap nanorods. Figure 4g shows the presence of calcium (Ca), phosphorus (P) and oxygen (O) elements in nanorods and the Ca/P molar ratio was estimated to be 1.6 (1 mole of calcium per 0.6 mole of phosphorus), which is equivalent to that of natural human bone (≈1.67) [48].

### Blood compatibility analysis

**Figure 5:**
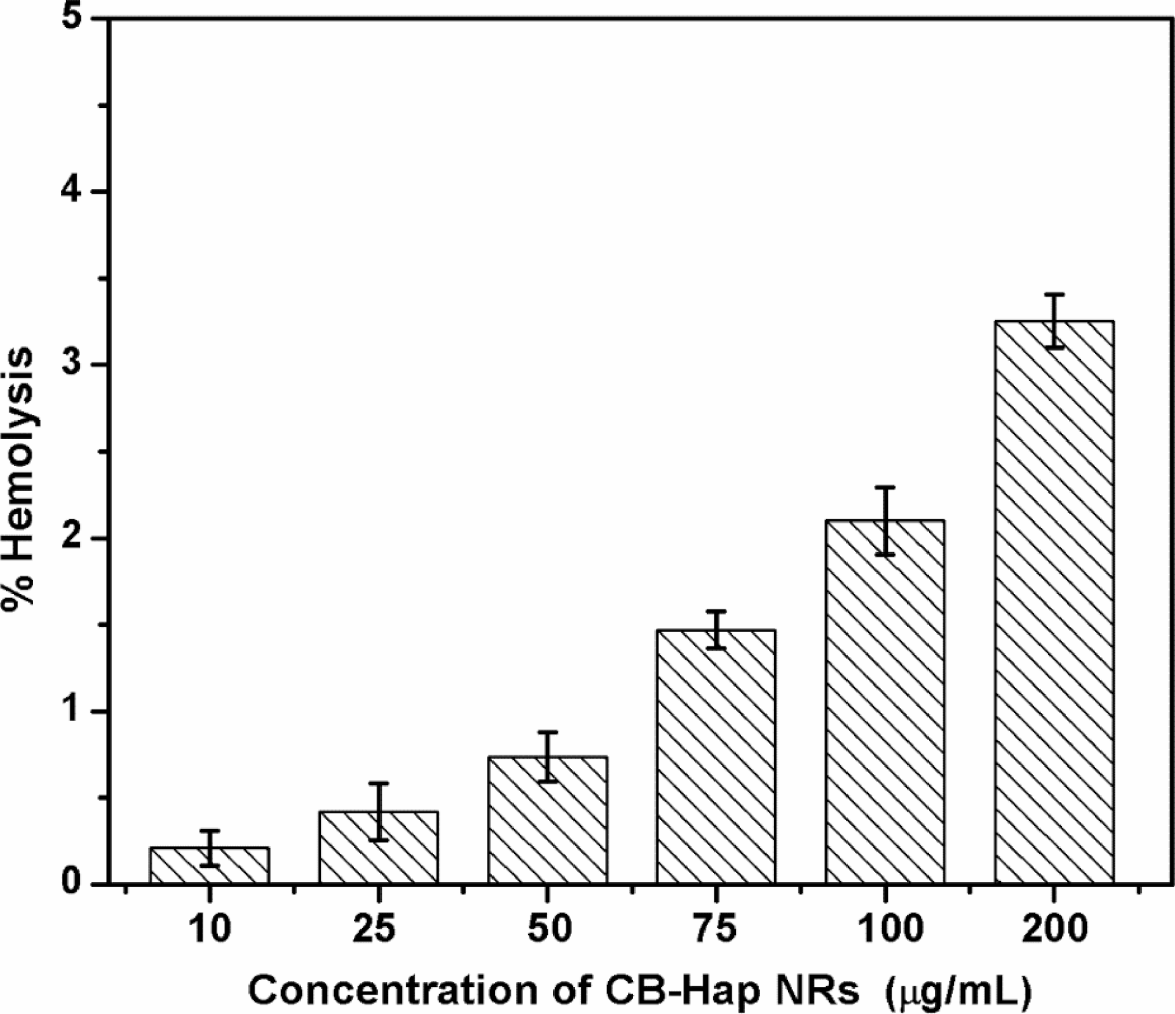
Hemolytic behavior of hydroxyapatite (Hap) nanorods using cuttlefish bone powder as a precursor (CB-Hap NRs) at various concentrations.

The hemolytic activity of the CB-Hap nanorods was investigated at various concentrations as shown in Figure 5 and the obtained results were calculated and compared with the American Society for Testing and Materials standard F756-00. Generally, the addition of hydroxyapatite microcrystals with blood will lead to hemolysis via aggregation of erythrocytes and membrane damage induced by crystals [49]. Moreover, Wiessner et al. (1988) showed that the crystallinity of the Hap plays a crucial role in determining the hemolytic properties [50]. However, the CB-Hap NRs exhibited less than 2% hemolysis up to 75 µg/mL, which indicates that the material is nonhemolytic and nontoxic to blood cells. Additionally, 2% and 3.2% hemolytic activity was recorded when a higher concentration of CB-Hap NRs, such as 100 and 200 µg/mL, respectively, was added to the blood samples. This indicates that the nanorods may exhibit a slight hemolytic behavior at a higher CB-Hap NR concentration. The hemolytic activity at higher concentrations can be attributed to the oxidative stress induced by introducing CB-Hap nanorods along with the blood cells [51]. The lower concentration of CB-Hap NRs may internalize into blood cells and degrade into ions which acts as a nutrient for the blood, whereas a high concentration of NRs may lead to an increase in the internal ions of the cells, elevating the internal pressure of the blood cells and inhibiting them [52]. Further, the length of the nanorods and their aggregation inside the blood vessels also may be the reason for its concentration dependent hemolytic activity [53]. Furthermore, Han et al. (2012) showed that the size and surface charge of Hap nanoparticles are responsible for hemolysis by aggregating red blood cells (RBCs) via bridging force mediated electrostatic interaction [54]. Thus, the hemolytic effect of CB-Hap NRs at high concentration can be reduced by optimizing their surface charge in the future.

### Antibacterial activity

**Figure 6:**
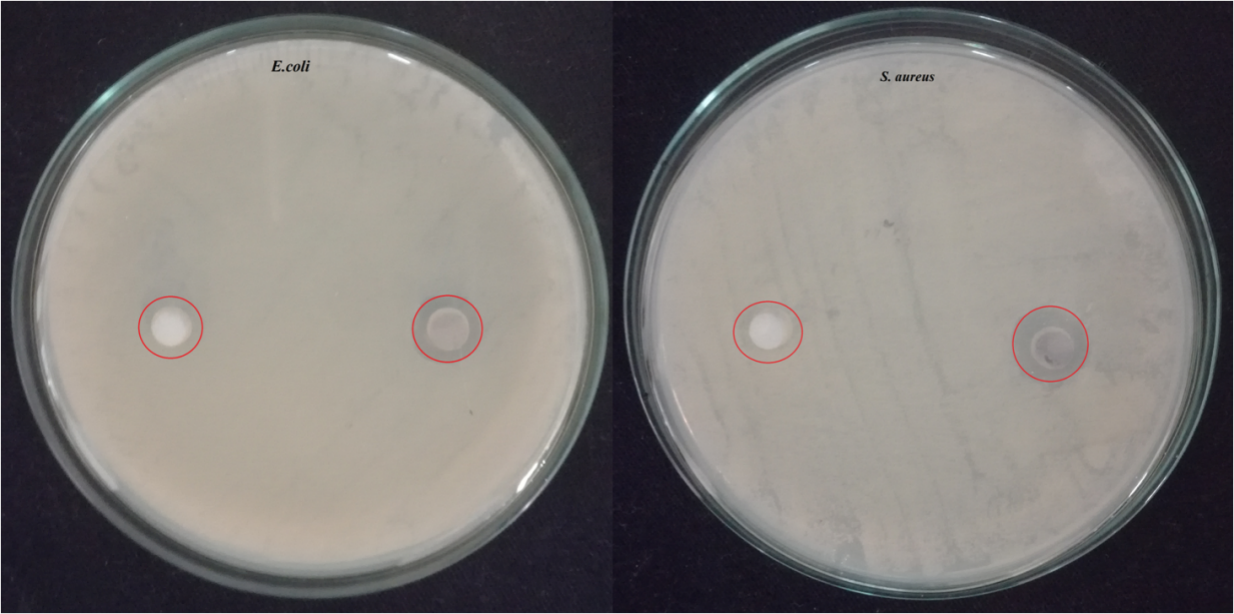
Antibacterial activity of hydroxyapatite (Hap) nanorods using cuttlefish bone powder as a precursor (CB-Hap NRs).

Generally, bacterial colonies can grow rapidly on Hap, due to their bioactive property and the presence of calcium and phosphate that act as nutrients for their growth. The formation of a biofilm (bacterial colonies) on Hap is one of the major causes of implant failure, therefore it is essential to study their bactericidal property. It can be noted from the literature that nanometer-sized Hap can effectively inhibit antibacterial activity but only when doped or cationic-substituted [55–56]. In contrast, the CB-derived Hap nanorods in the present study show optimum bactericidal effect on *E. coli* and *S. aureus* due to the size (>50 nm) and morphology of the material. However, no such activity was observed for CB alone. The obtained results are displayed in Figure 6 and the zone of inhibition in Table 1 shows a better bactericidal effect of Hap NRs towards *S. aureus* as compared with *E. coli*. This is due to the variations in cell structure, diffusion rate, metabolism and interaction of the nanoparticles with the microorganisms [57]. A similar study was conducted by Tank et al. (2014) to compare the antibacterial activity of pure and zinc-doped nanometer-sized Hap that are synthesized via a chemical precipitation mediated surfactant approach against gram positive *S. aureus*, *Bacillus cereus*, *Micrococcus luteus*, gram-negative *Pseudomonas aeruginosa* and *Shigella flexnari*. The study revealed that both pure and zinc-doped Hap have a needle-like morphology [58] and exhibited significant antibacterial activity against *M. luteus* and *S. aureus*, whereas moderate antibacterial activity was observed against *B. cereus* and *S. flexnari*. In addition, they also possess non-hemolytic activity with significant activity in simulated body fluid [59]. Hence, it is evident that the antibacterial activity of the present work can be improved via doping and the approach is highly safe to use for biological applications. This is important to emphasize as chemical synthesized nanoparticles may lead to toxic or side effects due to the existence of toxic chemicals [60]. The antibacterial mechanism of CB-Hap NRs may involve (i) the size-mediated penetration of nanorods into the bacterial cell wall to interact with the cellular biomolecules that increases the osmotic potential and its associated irreversible damage and (ii) the generation of free reactive oxygen species (ROS) radicals that are induced by nanorods that interact with the bacterial membrane and result in oxidative stress [61].

**Table 1:** Zone of inhibition of cuttlefish bone (CB) and hydroxyapatite (Hap) nanorods using cuttlefish bone powder as a precursor (CB-Hap NRs) towards gram-positive (*S. aureus*) and gram-negative (*E. coli*) bacteria.

Sample	Bacteria	Concentration (µg/mL)	Zone of inhibition (mm)

Hap Nanorods	*E. coli*	50	13 ± 0.5
Hap Nanorods	*S. aureus*	50	14.5 ± 0.5
CB	*E. coli*	50	–
CB	*S. aureus*	50	–

## Conclusion

This study is a pioneering work in the preparation of hydroxyapatite nanorods using marine waste cuttlefish bones using an oil-bath-mediated precipitation method. The resultant powders from the precipitation process were investigated via systematic characterization methods to confirm the physicochemical properties of the cuttlefish bone-derived nanorods. The XRD data revealed the mechanism behind the transformation of aragonite to calcite and then to Hap NRs of 20.86 nm to be related to crystallite size. The average length and width of the nanorods was determined as 79.05 and 219.66 nm using TEM analysis and the elemental analysis showed the Ca/P molar ratio of 1.6 (1 mole of calcium per 0.6 mole of phosphorus). Both the crystallite size and elemental analysis showed that the Hap NRs are similar to natural human bone and can be used as an implant material in hard tissue treatments. FTIR data further confirms the presence of phosphate groups and TGA data showed that the CB-Hap NRs possess significant thermal stability. Furthermore, the hemolysis study showed better blood compatibility than conventional, chemically synthesized Hap, and the antimicrobial activity showed better activity against gram-positive *S. aureus* bacteria*.* Moreover, the Hap can be incorporated with metal dopants in future experiments to further elevate their biocompatibility towards blood cells and increase their antibacterial efficacy. These enhanced biological properties of biogenic Hap NRs will be highly useful in the fabrication of novel implants for orthopedic and dental applications.

## Materials and Methods

The experiments were performed in a 1000 mL round bottom flask filled with calcium carbonate (CB powder), (NH_4_)_2_HPO_4_ and ammonia solution. The oil bath setup was used to maintain the reaction temperature. Silicone oil was used in the oil bath setup from 30 to 250 °C as it possesses high stability towards temperature and oxidation and can provide uniform temperature for long time, as compared to a water bath [62]. The color of the CB-Hap NRs turns light gray from white after annealing which may be due to the removal of organic products such as collagen and protein.

### Chemicals and reagents

Marine waste cuttlefish bones were collected as a source of calcium from Kasimedu fish market, which is located in Chennai, Tamil Nadu, India. All chemicals used for this study were of analytical grade such as (NH_4_)_2_HPO_4_ (Merck, purity – 99%, molecular weight (MW) – 132.06 g/mol), NH_4_OH (Merck, purity – 25%, MW – 35.05 g/mol), C_3_H_6_O (Sigma-Aldrich, purity – 99.8%, MW – 58.08 g/mol) and C_4_H_8_O_2_ (Sigma-Aldrich, purity – 99.9%, MW – 88.11 g/mol), CNa_2_O_3_ (Sigma-Aldrich, purity – 99.5%, MW – 105.99 g/mol), Na_3_C_6_H_5_O_7_ (Sigma-Aldrich, purity – 99%, MW – 258.07 g/mol) and phosphate buffer saline (PBS) (pH 7.4).

### Preparation of cuttlebone powder

The thick outer layer of the cuttlefish bone part, called the dorsal shield, was removed using a lancet and the inner part (lamellae matrix) was washed with distilled water, followed by acetone and ethanol to remove surface contaminants. The washed bones were dried in a hot air oven (Indfurr model OR-3795) at 60 °C for 24 h and 20 g of cuttlefish bone pieces were taken for top down processing using high-energy ball milling (VB Ceramic Consultants, Chennai) for 5 h. The ground bone powder was stored in a sterile plastic container at room temperature and placed in a desiccator for future experiments.

### Preparation of cuttlebone-Hap nanorods

**Figure 7:**
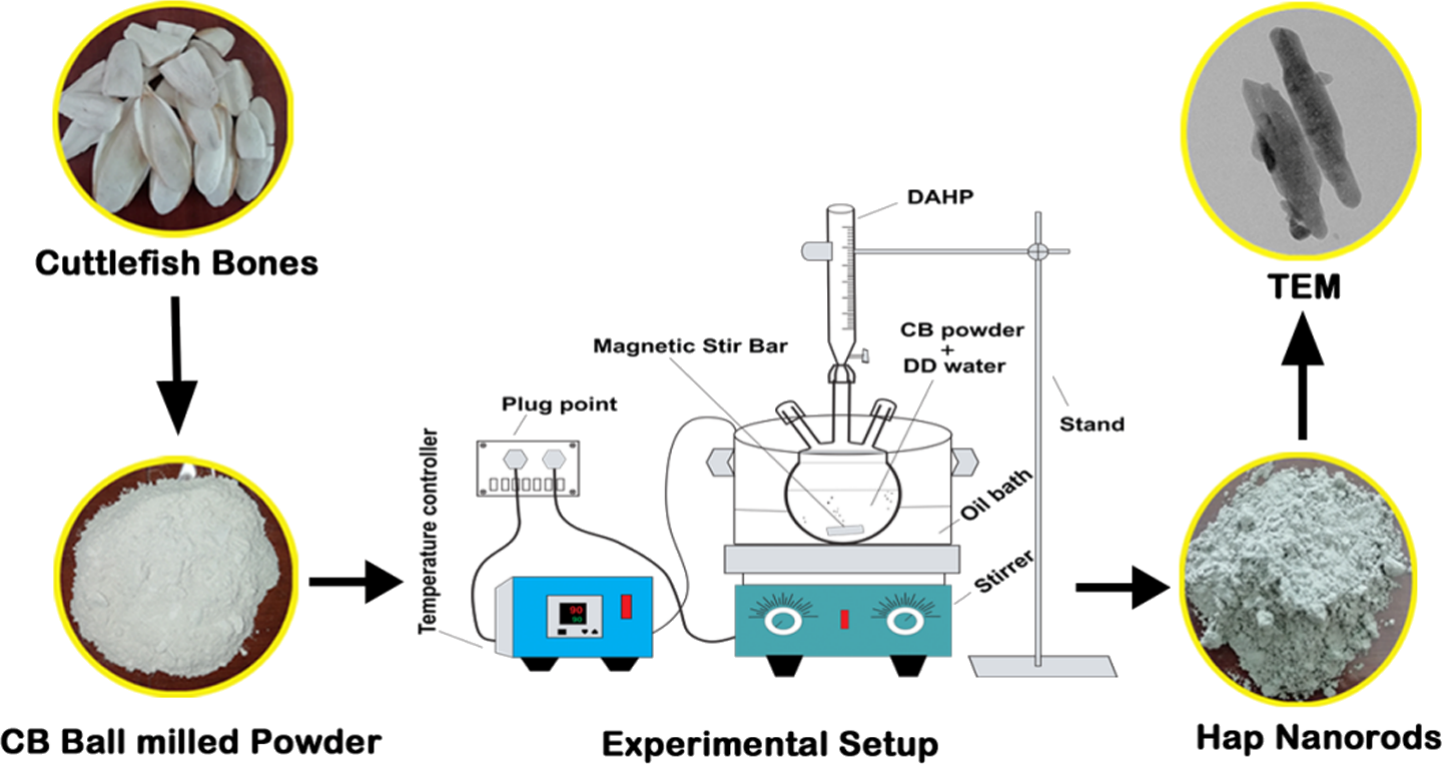
Oil-bath-mediated synthesis of CB-Hap NRs.

In the synthesis process, an oil bath was used as a temperature controller for the precipitation process and placed on magnetic stirrer. 1 molar (1.008 g) of cuttlefish bone powder was taken in a round bottom flask and 0.6 molar (0.795 g) of diammonium hydrogen phosphate was added dropwise using a burette. Then, the pH of the reaction was measured with a pH meter (Thermo Scientific, model-eco tester) and was adjusted from 8 to 12 using ammonia solution. This mixture was allowed to stir for various reaction times of 6, 12, 24, and 48 h at 80 °C and then the final precipitate was washed with distilled water and ethanol to remove impurities. Finally, the precipitate was dried at 60 °C in a hot air oven and was crushed into uniform powder using a mortar and pestle. The resultant powder was then annealed at 700 °C for 6 h and further characterized to confirm the presence of CB-Hap NRs as shown in Figure 7. The optimization of parameters such as reaction time, pH, temperature, and concentration plays a key role in the morphology evaluation of the nanomaterials. The parameters were optimized based on the study by Casella et al. (2017), in which Hap nanoparticles were synthesized with various morphologies such as rods, hexagonal prisms, hollow flower structure, and microspheres by adjusting the reaction time [63].

### Characterization of cuttlebone-Hap nanorods

#### X-ray diffraction

The X-ray diffraction (XRD) was carried out using an X-ray powder diffractometer (Bruker, Model D8 Advance) to study the crystallinity and phase formation of Hap nanorods. CB powder samples (ball milled) and CB-Hap NR powder was gently ground using a graphite mortar and pestle and placed on the sample holder. The crystallite size and phase formation of the cuttlefish-bone-derived Hap nanorods were identified and confirmed by comparing the data with standard XRD patterns from JCPDS.

#### Fourier transform infrared spectroscopy

The presence of functional groups in the cuttlefish bone and cuttlefish-bone-derived Hap nanorods was identified using a Perkin Elmer spectrum model Spectrum one FTIR spectrometer. CB powder (ball milled) and synthesized CB-Hap NR powders were directly placed on the sample holder. The sample was scanned from 4000 to 500 cm^−1^ in attenuated total reflectance (ATR) mode.

#### Transmission electron microscopy and energy dispersive X-ray analysis (EDX)

TEM micrographs of CB Hap NRs were obtained using TEM (model JEOL, Japan) at an operation voltage of 200 kV. A sufficient quantity of CB-Hap NR powder was placed on the carbon-coated copper grid and allowed to stick. The size and morphology of the synthesized nanohydroxyapatite was observed. The average particle size distribution of CB-Hap NRs was plotted using ImageJ software. The elemental composition analysis via energy dispersive X-ray analysis (EDX) was also performed to confirm the presence of CB-Hap NR conformations.

#### Thermal behavior analysis

Thermogravimetric analysis (TGA) was performed on raw cuttlebone and CB-Hap nanorods. 5 mg samples were placed on the sample holder and the thermal behavior was analyzed from room temperature to 700 °C using TGA model Q50 V20.13 Build 39, at a heating rate of 20 °C/min under 100 mL/min nitrogen flow rate.

### Biological studies

#### Antibacterial study

The bactericidal activity of the CB-Hap NRs was assessed using well diffusion methods against two different gram-positive and gram-negative bacteria, *E. coli*, and *S. aureus*, respectively*.* The nutrient broth with agar was autoclaved and transferred to a petri dish. Fresh bacterial cultures were spread over using a sterile cotton swab on solidified media. The CB-Hap NR samples were poured into the wells and were punctured in the nutrient agar media using micropipette tips. 50 µg/mL of free CB and CB-Hap NRs were introduced into the wells and the plates were incubated at 37 °C for 24 h, followed by zone of inhibition measurements. The method was repeated three times to check the reproducibility of the results.

#### Hemolysis assay

The experiments were conducted in accordance with the relevant laws and guidelines of Institutional Animal Ethics Committee (Tamil Nadu Veterinary and Animal Sciences University, Veppery, Chennai, India). The informed consent was obtained from the blood donor for the assay. Human blood was collected from a healthy volunteer with the help of a clinician and the blood was prevented from coagulation by mixing it with 3.2% of trisodium citrate (MW – 258.07 g/mol, purity – 99%) in the ratio 1:10. Various concentrations (200, 100, 75, 50, 25 and 10 µg/mL) of CB-Hap NRs were prepared with phosphate buffer saline (PBS) (purity – 99%, pH 7.4) and anticoagulated blood was used as a test specimen. 0.1% of sodium carbonate (MW – 105.99 g/mol, purity – 99.5%) with anticoagulated blood and anticoagulated blood with PBS were utilized as positive and negative controls, respectively. All of the test samples and controls were incubated at 37 °C for 3 h and then the tubes were centrifuged at 2000 rpm for 5 min. The optical density (OD) of the supernatant was recorded at 540 nm. All the tests were performed in triplicate to ensure reproducibility. The percentage of blood compatibility was calculated using the formula,


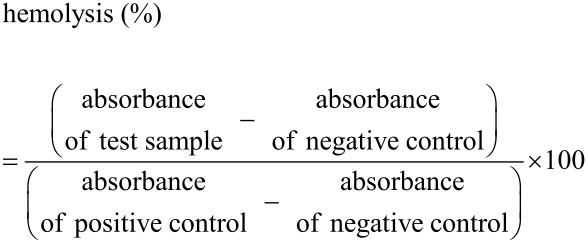


## Ethical approval

All procedures performed in the present study involving human participants are in accordance with the ethical standards of the Institutional Animal Ethics Committee (IAEC), Tamil Nadu Veterinary and Animal Sciences University, Chennai, India. Confidential informed consent was obtained from the participant (blood donor) included in the study.
